# Supplementary material for: Prediction of Putative Epitope Peptides against BaeR Associated with TCS Adaptation in Acinetobacter baumannii Using an In Silico Approach
Source: Medicina (Kaunas). 2023 Feb 11;59(2):343. doi: 10.3390/medicina59020343 (PMC9959147; doi:10.3390/medicina59020343)

Supplementary figure S1: Cluster analysis representing the functional relationships between the predicted peptides with a. MHC class I and b. MHC class II molecules represented by graphical tree and heat map formats with all the available alleles (Red zone indicates strong interactions and yellow zone indicates weak interactions)

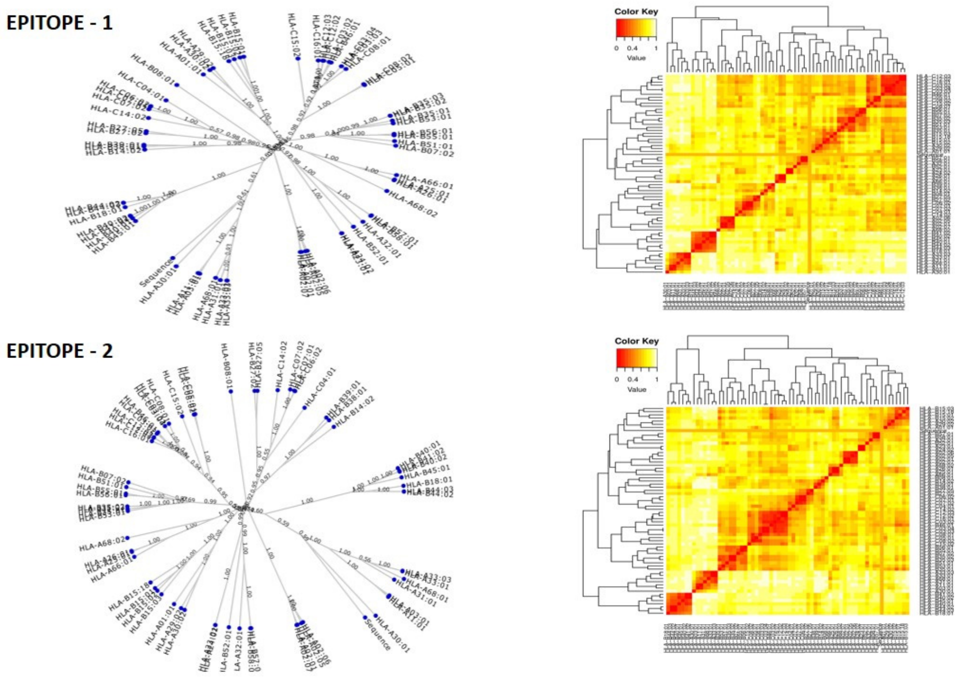

Supplement: Supplementary file 1 [file medicina-59-00343-s001.zip › medicina-2048883-Figure S1.pdf]
