# Supplementary material for: Prediction of Putative Epitope Peptides against BaeR Associated with TCS Adaptation in Acinetobacter baumannii Using an In Silico Approach
Source: Medicina (Kaunas). 2023 Feb 11;59(2):343. doi: 10.3390/medicina59020343 (PMC9959147; doi:10.3390/medicina59020343)
Supplement: Supplementary file 1 [file medicina-59-00343-s001.zip › medicina-2048883-Table S1.pdf]

**Supplementary Table S1: Consensus method predictions with HLA alleles for T-cell MHC class-I and class-II binding epitopes based on the percentile ranks (Lowest rank) and $IC_{50}$ values (<50nM – High binding affinity)**

| *Peptide sequences* | *Alleles* | *Start* | *End* | *Length* | *Peptide* | *Percentile rank* | *ANN $IC_{50}$* | *ANN rank* | *SMM $IC_{50}$* | *SMM rank* | *Comb Lib score* | *Comb Lib rank* | *Net MHC pan score* | *Net MHC pan rank* |
|---|---|---|---|---|---|---|---|---|---|---|---|---|---|---|
| **T cell MHC class I HLA alleles and binding peptides** | | | | | | | | | | | | | | |
| RKVREQSDLPIIMVTARTEEIDRVLGLNMGADDYVC | HLA-B*40:01 | 4 | 12 | 9 | REQSDLPII | 0.29 | 74.55 | 0.19 | 55.45 | 0.4 | - | - | - | - |
| | HLA-B*44:03 | 19 | 27 | 9 | EEIDRVLGL | 0.41 | 296.13 | 0.51 | 177.6 | 0.3 | - | - | - | - |
| | HLA-B*35:01 | 9 | 17 | 9 | LPIIMVTAR | 0.47 | 199.09 | 0.47 | 192.6 | 2.4 | 8.51E-06 | 0.2 | - | - |
| | HLA-A*68:02 | 13 | 21 | 9 | MVTARTEEI | 0.5 | 13.41 | 0.14 | 38.6 | 0.5 | 0.000112 | 5.7 | - | - |
| | HLA-B*40:01 | 18 | 27 | 10 | TEEIDRVLGL | 0.52 | 494.94 | 0.74 | 89.54 | 0.3 | - | - | - | - |
| HVGQVYSRAQLLDHINPDSFDV | HLA-A*24:02 | 5 | 13 | 9 | VYSRAQLLD | 0.95 | 875.91 | 1.3 | 300.3 | 0.6 | - | - | - | - |
| | HLA-B*15:01 | 11 | 20 | 10 | LLDHINPDSF | 1.55 | 346.43 | 1.4 | 23.46 | 1.7 | - | - | - | - |
| | HLA-B*40:01 | 3 | 11 | 9 | GQVYSRAQL | 1.76 | 593.58 | 0.83 | 847.0 | 2.7 | - | - | - | - |
| | HLA-A*24:02 | 5 | 14 | 10 | VYSRAQLLDH | 2.35 | 3186.63 | 3 | 790.1 | 1.7 | - | - | - | - |
| | HLA-A*01:01 | 11 | 20 | 10 | LLDHINPDSF | 2.45 | 4711.83 | 2.5 | 2623.0 | 2.4 | - | - | - | - |
| **T cell MHC class II HLA alleles and binding peptides** | | | | | | | | | | | | | | |
| RKVREQSDLPIIMVTARTEEIDRVLGLNMGADDYVC | HLADRB3*01:01 | 8 | 22 | 15 | DLPIIMVTARTEEID | 11 | 404.2 | 11 | 5358 | 48 | 0.01 | 0.01 | - | - |
| | HLA-DRB3*01:01 | 10 | 24 | 15 | PIIMVTARTEEIDRV | 11 | 418.3 | 11 | 2553 | 29 | 0.01 | 0.01 | - | - |
| HVGQVYSRAQLLDHINPDSFDV | HLA-DRB4*01:01 | 2 | 16 | 2 | VGQVYSRAQLLDHIN | 11 | 171.1 | 11 | 190 | 4.5 | 1931.06 | 50 | - | - |
| | HLA-DRB4*01:01 | 3 | 17 | 3 | GQVYSRAQLLDHINP | 12 | 150.9 | 9.1 | 395 | 12 | 1931.06 | 50 | - | - |
| | HLA-DRB4*01:01 | 1 | 15 | 1 | HVGQVYSRAQLLDHI | 12 | 181.9 | 12 | 191 | 4.5 | 1931.06 | 50 | - | - |
| | HLA-DRB4*01:01 | 4 | 18 | 4 | QVYSRAQLLDHINPD | 12 | 176.6 | 11 | 383 | 12 | 42.91 | 26 | - | - |
